# The Shady Pink Elephant: End of Life Education for Young Women Affected by Breast Cancer

**DOI:** 10.1007/s13187-018-1446-1

**Published:** 2018-12-27

**Authors:** Jean Rowe, Tara Schapmire

**Affiliations:** 1 Young Survival Coalition, 75 Broad Street, Suite 409, New York, NY 10004 USA; 2 Interdisciplinary Program for Palliative Care & Chronic Illness, University of Louisville School of Medicine, 501 East Broadway, Suite 330, Louisville, KY 40202 USA

**Keywords:** Breast, Metastatic, End of life, Young

## Abstract

In an effort to improve participation of younger breast cancer survivors in end of life (EOL) discussions and planning, this study evaluated the impact of The Shady Pink Elephant EOL educational series on participants’ knowledge, attitudes and behaviors towards palliative care and EOL wishes. Data was gathered at baseline (pre survey and registration) following each event (post survey) and 6 months after the series as intervention (post survey). A total of 36 women with breast cancer, averaging 40 years of age, participated in the first online event, 24 in the second and 22 in the third. A total of 20 completed the 6-month post survey. Significant improvement in scores occurred from baseline to 6 months for the following items: belief that palliative care is only for those at the EOL, belief that EOL discussions are only important for those at the EOL, comfort with talking about EOL issues, confidence that EOL wishes will be honored by one’s health care power of attorney and knowledge of characteristics are important in the person assigned as a person’s health care power of attorney. The Shady Pink Elephant EOL educational series is therefore a promising intervention for improving EOL knowledge, attitudes and behavior. Further research with larger sample sizes is needed regarding understanding and accessing palliative care and deciding upon and communicating EOL wishes in this patient population.

## Introduction and Background

In the USA, breast cancer is the most common malignant tumor in young women ages 15 to 39 [1]. Women under age 40 account for 7% of breast cancer diagnoses in the USA [2] with over 11,000 cases of breast cancer expected in women under age 40 in 2018 [3]. Since 1976 in the USA, distant disease in women under age 39 has steadily increased [1], and nearly 1000 women under age 40 die from breast cancer every year [3]. End of Life (EOL) planning and discussions are important components of care for breast cancer patients but are not standard practice in healthcare programs [4]. Metastatic breast cancer (MBC) study participants have reported having neither prepared written advance directives nor talking with loved ones about their wishes [4]. Informal discussions about EOL decisions take place more frequently with friends and family than with physicians [4]. Younger patients with advanced cancer have reported feeling less prepared for EOL [5]. Studies have reported on suggested interventions for improving EOL knowledge, but the majority of these samples are comprised of older adults [6–10]. According to the Institute of Medicine, people who participate in palliative or hospice care might live longer than those who do not [11]; nevertheless, referrals to palliative care which can result in advance care planning and EOL discussions are slow to happen. Patients state they would like EOL care that alleviates pain and suffering, but the reality is that they often receive acute care from physicians who may not be as familiar with them [11]. Younger, poor, minority and less educated individuals often do not have clinician-patient conversations about their EOL care values, goals and preferences, one goal of which is to avoid unwanted treatment [11]. Strategies are needed to diminish communication barriers between physicians and seriously ill patients and their families regarding EOL. In response to constituent feedback, Young Survival Coalition (YSC) aimed to improve younger breast cancer survivors’ (those diagnosed at the age of 40 or younger) knowledge, attitudes and behaviors regarding understanding and accessing palliative care as well as deciding and communicating (EOL) wishes. YSC is dedicated to the critical issues unique to young women who are diagnosed with breast cancer. It offered education and tools regarding EOL striving to remove stigma and allow for EOL discussions and advance care planning. The objectives were (1) to implement the EOL education series for young women affected by breast cancer and (2) to evaluate the impact of the curriculum on participants’ knowledge, attitudes and behavior regarding understanding and accessing palliative care as well as deciding upon and communicating EOL wishes. From December 2015 through September 2016, The Shady Pink Elephant EOL study was implemented. We utilized a non-experimental, pre/post survey design with surveys at baseline, after each online event and 6 months after the last online event.

### Methods

This study included a non-probability sample of English-speaking women who were diagnosed with any stage of breast cancer at the age of 41 or younger and who were between the ages of 18 and 50 years at the time of the study. Those not meeting these criteria were not included in this study. While 68 individuals met criteria, a total of 36 individuals participated in the first online event and completed the post survey. Twenty-four completed the post survey in the second online event, and 22 completed the post survey in the third online event. A total of 20 completed the 6-month post survey.

### Procedure

The EOL series was advertised through all of YSC’s social media platforms, e-blasts and shared with other breast cancer organizations and partners. A short list includes SHARE, Living Beyond Breast Cancer, Sharsheret, the Metastatic Breast Cancer Alliance, Stupid Cancer, the Dana Farber Cancer Institute and Johns Hopkins Medical Institute. The study was approved by the University of Louisville Human Subjects Protection Program. The invitation to participate in the study was included in the email confirming registration for the online event with a link to the online pre survey. Six months following the entire series, a follow-up email with a link to the 6-month post survey was distributed to participants who completed the pre survey and all three parts of the educational series including post surveys after each event. Only data provided by those consenting to the research are included here.

### The Intervention

The *Young Survival Coalition’s Shady Pink Elephant: End of Life Education Series for Women Affected by Breast Cancer* was a three part online series of interactive, live-streaming events about EOL topics. The objectives for the three part educational event are highlighted in Table 1. The first online event focused on the research on the benefits of introducing palliative care at the beginning of or earlier in a cancer survivor’s journey regardless of stage. The second online event focused on the * Let's Have Dinner and Talk about Death* model in which the founder of this grass roots movement discussed initiating EOL discussions and social action over dinner. The third online event focused on the legal decisions and documents suggested to have in place for EOL planning. Each online event was 60 minutes in length. The event was delivered through YSC’s Facebook page as a live video event. Numbers of people who attended the live event were captured at the time. Participants needed to be able to have access to Facebook to participate. They then could watch and listen to the presenter and type in questions during the live event. All three events were recorded and are housed on YSC’s YouTube channel. That platform captures numbers of views of each event.

**Table 1:** Shady Pink Elephant EOL education series

Session topic		
Research and Benefit of Introducing Palliative Care Early	Let's Have Dinner and Talk about Death	The Nuts and Bolts of End of Life Planning
Objectives		
1. Articulate the purpose of palliative care and the ways in which it differs from hospice care.	1. Learn about a grassroots movement to initiate end of life discussions and social action.	1. Identify one or more reasons why it is important for young adults with cancer to have a valid advance health care directive in place
	2. Detect cultural myths that people are afraid to have end of life discussions.	
2. Identify at least 2 medical benefits of early initiation of palliative care for young adults with cancer		
		2. List the individual parts that make up an advance health care directive
	3. Improve awareness that end of life discussions are available and important for all people at any time and not just for individuals with advance cancer or at the end of life.	
		3. Identify one or more characteristics that young adults with cancer should look for in an agent
3. List at least 2 psychosocial benefits of early initiation of palliative care for young adults with cancer		
	4. Identify ways to support those closest to us who are facing end of life and to discuss with them how they would like to live their final days	

### Measures

Pre and post surveys were developed by the researchers in conjunction with the series presenters and included questions specific to the learning objectives of each part in the series. They included 12 true/false or yes/no questions and 11 Likert-scaled statements (1 being strongly disagree and 7 being strongly agree) exploring knowledge, attitudes, and behaviors related to EOL issues. All survey questions are listed in Tables 3 and 4.

### Statistical Analysis

Demographic information was summarized by frequencies and percentages. Paired sample *t* tests were used to assess pre and post score differences of the items with the Likert-scaled response formats. Examination of the pre and post score differences shows that the assumption of normality for all items was met. Cohen’s *d* was calculated to assess the effect size of these differences; an effect of .20 is considered small, .50 is moderate, and .80 is large [12]. McNemar’s chi exact tests were used for nominal data. Statistical significance was set by convention at *p* < 0.05. All analyses were performed using IBM SPSS V. 24.

## Results

Thirty-six series participants consented and completed the baseline survey before the first online event. Of those, 20 participated in the survey at 6 months post the last online event. Table 2 highlights the demographics at baseline. The women averaged 40 years of age, 58% had stage I or II breast cancer, and 56% were in active treatment. Reasons for attrition included death (1 participant), completed the pre event registration survey but did not participate in any of the three online events (10 participants), did not participate in the second or third online events (3 participants), and did not participate in the third online event (2 participants).

**Table 2:** Demographic Characteristics of Participants at Baseline (*N* = 36)

Characteristic	*n* (%)	M (SD)	Range
Age in years		40.4 (0.50)	27–52
Breast cancer stage			
I	10 (27.8)		
II	11 (30.6)		
III	4 (11.1)		
IV	9 (25.0)		
Unknown	2 (5.6)		
Currently in Active Treatment	20 (55.6)		
Residency			
Urban	14 (38.9)		
Suburban	16 (44.4)		
Rural	5 (13.9)		

Table 3 highlights the proportion of preferred/correct responses to the categorical (true/false, yes/no) knowledge, belief and behavior questions from baseline to 6-month post survey. All but two (*I am currently receiving palliative care as part of my treatment plan* and *I plan to establish my health care power of attorney in the near future*) had increases in the proportion of preferred/correct responses. Only the question asking participants if they had discussed their EOL plans with co-survivors (e.g. family, friends, loved ones) had a significant difference (*p* < 0.05) in the proportion of preferred responses from baseline to 6-month post survey while the question asking participants if they understood the individual parts of an advance directive trended towards significance (*p* < 0.10).

**Table 3:** Prevalence of Correct/Preferred Responses to Categorical End of Life Series Knowledge, Belief and Behavior Questions from Baseline to 6-month Post Survey

Item	Preferred Response	Correct/Preferred Responses	
		Baseline (*N* = 36)	Six Months (*N* = 20)
		*n* (%)	*n* (%)
There is no difference between palliative and hospice care.	False	29 (82.9)	19 (95.0)
Someone on my healthcare team has discussed palliative care with me.	Yes	5 (14.3)	3 (15.0)
I am currently receiving palliative care as part of my treatment plan.	Yes	4 (11.4)	2 (10.0)
I have discussed my end of life plans with my health care providers.	Yes	4 (11.4)	4 (20.0)
**I have discussed my end of life plans with my co-survivors (family, friends, loved ones).**	Yes	21 (60.0)	17 (85.0)*
I have told at least one person how I would like to live my final days.	Yes	21 (60.0)	15 (75.0)
If the setting was warm, authentic and trusting, I would talk about my wishes for end of life.	Yes	34 (94.4)	19 (95.0)
I know what an advance healthcare directive is.	Yes	25 (71.4)	20 (100.00)
**I understand the individual parts of an advance directive.**	Yes	16 (45.7)	16 (80.0)~
I know what a healthcare power of attorney agent is.	Yes	31 (88.6)	19 (95.0)
I have an advance directive.	Yes	13 (37.1)	11 (55.0)
I plan to create an advance directive in the near future.	Yes	11 (37.9)	7 (43.8)
I have a healthcare power of attorney.	Yes	19 (54.3)	14 (70.0)
I plan to establish my health care power of attorney in the near future.	Yes	10 (34.5)	4 (33.3)

Note: McNemar’s Exact tests for significance used. ~*p* < 0.10; *p < 0.05

Table 4 highlights the changes in means from baseline to 6-month post survey on the Likert scaled knowledge, belief and behavior questions. All normality assumptions were met for the Likert scaled items. Means improved on all items. Significant improvement in means from baseline to 6 months occurred for the following items: belief that palliative care is only for those at the EOL (*t* = 2.56, *df* = 18, *p* < 0.05) and belief that EOL discussions are only important for those at the EOL (*t* = 2.64, *df* = 18, *p* < 0.05). For both of these items, levels of disagreement were greater after intervention with moderate effect sizes (Cohen’s *d* = 0.59 and Cohen’s *d* = 0.61 respectively). Additional significant improvement in means (increasing levels of agreement) occurred for: comfort with talking about EOL issues (*t* = − 2.14, *df* = 18, *p* < 0.05); confidence that my EOL wishes will be honored by my health care power of attorney (*t* = − 2.11, *df* = 18, *p* < 0.05) and knowledge of characteristics important in the person I assign as my healthcare power of attorney (*t* = − 3.08, *df* = 18, *p* < 0.01). Effects sizes were moderate (Cohen’s *d* > 0.50) for these items with the exception of the item addressing confidence that my EOL wishes will be honored by my health care power of attorney; this effect size was small (Cohen’s *d* = 0.49).

**Table 4:** Mean Differences between Baseline and 6-month Post Test End of Life Series Questions

Scaled Item (1 being strongly disagree and 7 being strongly agree)	Baseline (*N* = 36)		Six Months (*N* = 20)		*t(df)*	Cohen’s *d*
	M	SD	M	SD		
I would feel comfortable talking about palliative care with my healthcare provider.	5.74	1.28	5.95	1.35	− 0.443(18)	0.10
I would not feel comfortable talking about palliative care with my co-survivors (family, friends, loved ones).	2.68	1.63	1.95	1.18	1.55(18)	0.36
I believe palliative care is an important part of cancer treatment.	6.26	1.15	6.47	1.39	− 0.47(18)	0.11
**I believe that palliative care is only for those at the end of life.**	2.37	1.46	1.47	1.02	2.56(18)*	0.59
I believe I could benefit from palliative care as part of my cancer treatment.	4.74	1.85	4.89	2.21	− 0.39(18)	0.09
**I believe that end of life discussions are only important for those at the end of life.**	3.37	2.31	1.79	1.27	2.64(18)*	0.61
I believe it is important that everyone affected by cancer discuss their end of life wishes with their healthcare providers regardless of the stage of their illness.	5.89	1.52	5.95	1.54	− 0.18(18)	0.42
**I am comfortable talking about end of life issues**.	5.58	1.35	6.12	0.99	− 2.14(18)*	0.50
If I have communicated end of life wishes, I feel confident that they will be honored by my healthcare providers.	4.94	1.59	5.44	1.20	− 1.49(17)	0.35
**If I have communicated end of life wishes, I feel confident that they will be honored by the person I assign as my healthcare power of attorney agent.**	5.21	1.58	5.95	1.18	− 2.11(18)*	0.49
**I know what characteristics are important in the person I may assign as my healthcare power of attorney agent.**	5.00	1.67	6.42	0.77	− 3.08(18)**	0.71

Note: ~*p* < 0.10; *p < 0.05; **p < 0.01. For all measures, higher means indicate higher levels of agreement

## Discussion

The main objective of our study was to evaluate the impact of the YSC EOL educational series on participants’ knowledge, beliefs and behaviors regarding understanding and accessing palliative care as well as deciding upon and communicating EOL wishes. While all knowledge, beliefs and behaviors on EOL scores tended to improve from baseline to 6-month post survey, not all were statistically significant. Participants were significantly more likely to discuss their EOL wishes with co-survivors 6 months after the intervention. They also reported a significantly better understanding of the individual parts of an advance directive 6 months after the intervention. Beliefs about palliative care and the importance of having early EOL discussions also significantly improved 6 months after the intervention. Comfort levels around talking about EOL issues also significantly improved 6 months after the intervention. Participants’ confidence that their wishes would be honored by their healthcare power of attorney significantly improved at 6-month post intervention, as well as their reported knowledge of the important characteristics of these assigned agents.

The strength of this study is its focus on younger women living with breast cancer. Limitations include the small pool of participants, attrition challenges and lack of validated, standardized measures of outcomes. Future studies could target larger participant groups by studying young adults with any cancer diagnosis rather than limiting it to young women living with breast cancer to obtain more robust results.

## Conclusion

The Shady Pink Elephant EOL educational series is a promising intervention for improving young breast cancer survivors’ EOL knowledge, attitudes and behavior regarding understanding and accessing palliative care as well as deciding and communicating EOL wishes. Further research with a larger patient population is needed regarding young cancer patients’ views and experiences with palliative care and EOL planning; access to that information may need more global marketing efforts and grassroots sharing.
